# Small Bed-Chambers

**Published:** 1868-08

**Authors:** 


### SMALL BED-CHAMBERS.

THERE is reason to believe that more cases of dangerous and fatal disease are gradually engendered annually by the habit of sleeping in small, unventilated rooms, than have occurred from a cholera atmosphere during any year since it made its appearance in this country. Very many persons sleep in eight by ten rooms, that is, in rooms the length and breadth of which multiplied together, and this multiplied again by ten for the height of the chamber, would make just eight hundred cubic feet, while the cubic space for each bed, according to the English apportionment for hospitals, is twenty-one hundred feet. But more, in order "to give the air of a room the highest degree of freshness," the French hospitals contract for a complete renewal of the air of a room every hour, while the English assert that double the amount, or over four thousand feet an hour, is required. Four thousand feet of air every hour! and yet there are multitudes in the city of New-York who sleep with closed doors and windows in rooms which do not contain a thousand cubic feet of space, and that thousand feet is to last all night, at least eight hours, except such scanty supplies as may be obtained of any fresh air that may insinuate itself through little crevices by door or window, not an eighth of an inch in thickness. But when it is known that in many cases a man and wife and infant sleep habitually in thousand-feet rooms, it is no marvel that multitudes perish prematurely in cities; no wonder that infant children wilt away like flowers without water, and that five thousand of them are to die in the city of New-York alone during the hundred days which shall include the fifteenth of July, eighteen hundred and sixty! Another fact is suggestive, that among the fifty thousand persons who sleep nightly in the lodging-houses of London, expressly arranged on the improved principles of space and ventilation already referred to, it has been proven that not one single case of fever has been engendered in two years! Let every intelligent reader improve the teachings of this article without an hour's delay.

